# Preparation and Properties of F-Doped PrBa_0.8_Sr_0.2_Co_2_O_5+δ_ Perovskite Cathode Materials

**DOI:** 10.3390/molecules30051140

**Published:** 2025-03-03

**Authors:** Mengxin Li, Songbo Li, Shengli An, Ning Li, Runze Sun, Yuanyuan Ma, Hongli Qiao, Yanpeng Liu, Xu Zhang

**Affiliations:** 1 School of Chemistry and Chemical Engineering, Inner Mongolia University of Science and Technology, Baotou 014010, China; 15175447968@163.com (M.L.); 18614847792@163.com (N.L.); 18264331178@163.com (R.S.); m13964589510@163.com (Y.M.); 18434836609@163.com (H.Q.); liu126263301@163.com (Y.L.); 15665700619@163.com (X.Z.); 2 School of Rare Earth Industry, Inner Mongolia University of Science and Technology, Baotou 014010, China; san@imust.edu.cn

**Keywords:** solid oxide fuel cell, F doping, cathode material, electrochemistry

## Abstract

F-doped PrBa_0.8_Sr_0.2_Co_2_O_5+δ−x_F_x_ (PBSCF_x_, *x* = 0, 0.025, 0.05, 0.075, 0.1) cathode powder was synthesized by the sol–gel method. X-ray diffraction results showed that all the samples doped with F exhibited a typical tetragonal perovskite structure without a heterophase. F doping can effectively reduce the thermal expansion coefficient (TEC) of the cathode materials, which decreased from 25.3699 × 10^−6^ K^−1^ of PBSC to 23.5295 × 10^−6^ K^−1^ of PBSCF_0.1_. The area-specific resistance (ASR) of PBSCF_0.05_ was 0.0207 Ω·cm^2^ at 800 °C, with a conductivity of 1637.27 S·cm^−1^ and maximum power density of 856.08 mW·cm^−2^. Its performance had slight decay after 100 h at 800 °C. F doping significantly improved the electrochemical performance of this cathode material for solid oxide fuel cells (SOFCs).

## 1. Introduction

A SOFC is a kind of direct electrochemical energy conversion device in all solid-state working conditions. As a result of its advantages of high energy efficiency, zero pollution, and strong fuel adaptability, SOFCs exhibit extensive potential in energy conversion and are of significant interest to researchers [1,2,3]. SOFCs can generally maintain excellent cell performance at high operating temperatures, but the durability and service life will be greatly reduced [4]. The reduction in operating temperature usually leads to a sharp increase in the cathode polarization resistance, thereby reducing cell performance [5]. Therefore, finding a cell material that can maintain good performance and stability under medium- and low-temperature conditions has become one of the key points in the current development of SOFCs.

At present, perovskite has an advantage in research due to its wide compositional and crystal phase variability, and its physical and chemical properties can be regulated by cation substitution at the A and B lattice sites [6]. Double-perovskite materials can exhibit better activity, stability, and efficiency than perovskite, so it is necessary to change from single perovskites to double perovskites to achieve improved catalytic performance of the materials [7]. Double-perovskite materials LnBaCo_2_O_5+δ_ (Ln = Pr, Gd, Sm) have been widely studied due to their high mixed ion–electron conductivity [8,9]. LnBaCo_2_O_5+δ_ is alternately composed of Ln–O|Ba–O|Co–O layers along the C-axis, in which a large number of oxygen vacancies facilitate oxygen ion transport [10]. Among them, PrBaCo_2_O_5+δ_ exhibits high electronic and ionic conductivity in the medium-temperature range. Furthermore, it possesses a high oxygen surface exchange and volume diffusion coefficient, demonstrating excellent performance as the cathode of SOFCs. However, it exhibits an elevated TEC compared with conventional electrolyte materials [11]. Therefore, TEC must be minimized while maintaining superior electrochemical performance. Partial substitution or defect methods are commonly employed to improve the electrochemical properties of cathodes and reduce TEC. Reports indicate that the partial substitution of Ba^2+^ with Ca^2+^/Sr^2+^ in LnBaCo_2_O_5+δ_ enhances the electrochemical properties of the material. Moreover, it exhibits a certain inhibitory effect on the enhancement of TEC [12,13]. According to Yang et al. [14], after Ca^2+^ partially replaces Sm^3+^ and Ba^2+^, Co^3+^ can be limited to the intermediate spin state, which reduces lattice expansion, effectively decreasing the TEC of the material and exhibiting good electrochemical performance in Sm_0.8_Ca_0.2_Ba_1−*x*_Ca*_x_*Co_2_O_5+*δ*_ cathode materials. Dong et al. [15] found that the partial substitution of Ba^2+^ by Sr^2+^ in Pr_0.94_BaCo_2_O_5+δ_ significantly improves the charge transfer ability and reduces the activation energy of oxygen adsorption. Wu et al. [16] proved that the partial substitution of Ba^2+^ by Sr^2+^ in LnBaCo_2_O_5+δ_ disturbs the arrangement order of Pr-O and Ba-O layers, and this disturbance can increase the content of Co^4+^, improving electrochemical performance and oxygen reduction reaction (ORR) activity. Partial substitution of O^2−^ by F^−^ and Cl^−^ is also considered feasible [17,18]. Xu et al. [19] reported that the partial substitution of Cl^−^ promotes the formation of oxygen vacancy and accelerates the migration of O^2−^, and appropriate substitution of Cl^−^ for O^2−^ shows good ORR activity, CO_2_ tolerance, and operational stability. Zhang et al. [20] reported that F-doped Sr_2_Fe_1.5_Mo_0.5_O_6−δ_ promotes the diffusion and surface adsorption of oxygen at the cathode by weakening the chemical bond between metal cation and O^2−^, significantly improving the reaction kinetics of the rate-limiting step. However, the effect of anion partial substitution on the TEC of cathode materials has been inadequately examined.

Therefore, this study proposes the use of F-partial substitution of O^2−^ to improve the electrochemical properties of PrBa_0.8_Sr_0.2_Co_2_O_5+δ_ cathode materials and explore the effect of F-partial substitution on TEC.

## 2. Results and Discussion

**Figure 1:**
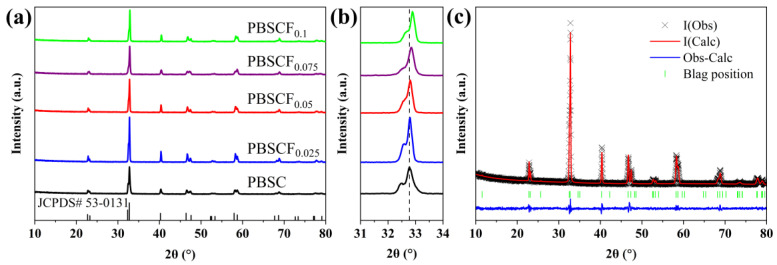
(**a**) XRD patterns of the cathode material PBSCF_x_; (**b**) localized magnification; (**c**) Rietveld refinement pattern of the PBSCF_0.05_ sample.

Figure 1a shows the XRD patterns of the PBSCF_x_ (*x* = 0, 0.025, 0.05, 0.075, 0.1) series cathode materials. The absence of an impurity phase following the partial substitution of O by F indicated that PBSCF_x_ was a pure phase, showing a well-crystallizable quartet phase structure (JCPDS No.53-0131). The space group was P4/mmm [15]. (b) The local amplification. With the increase in F, the XRD peak of PBSCF_x_ gradually shifted to a higher angle and the cell volume gradually decreased, which was caused by the smaller ionic radius of F^−^ (1.33 Å) than that of O^2−^ (1.4 Å) [20]. (c) The refined patterns of PBSCF_0.05_, and the results showed that the fitting results were consistent with the test results. The refined results of the PBSCF_x_ series cathode materials are shown in Table 1. As the doping amount of F increased, the cell parameters gradually decreased, which was consistent with the XRD results.

**Figure 2:**
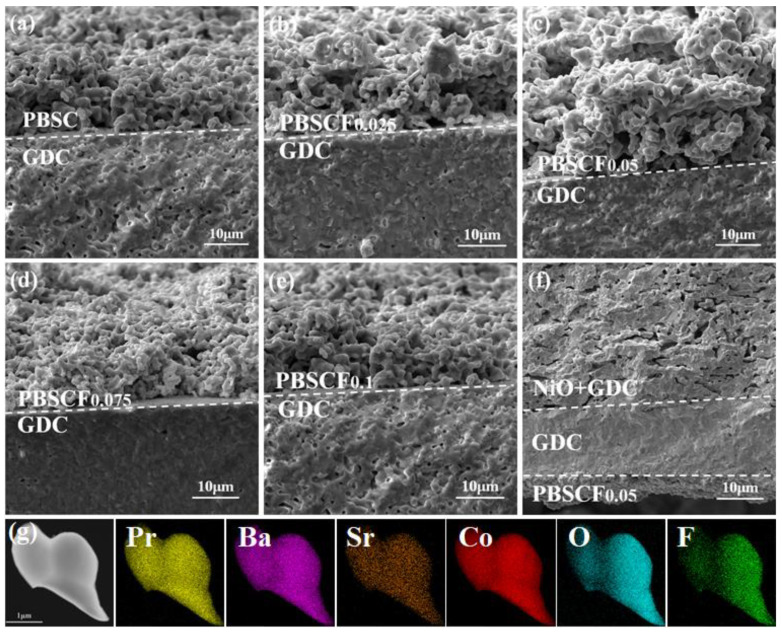
(**a**–**e**) SEM of PBSCF_x_|GDC|PBSCF section; (**f**) SEM of NiO + GDC|GDC|PBSCF_0.05_ section; (**g**) TEM image and EDS maps of the PBSCF_0.05_ powder.

Figure 2 shows the cross-section SEM of the PBSCF_x_ series cathode materials uniformly brushed on both sides of the GDC electrolyte by screen printing technology to prepare PBSCF_x_|GDC|PBSCF_x_ symmetrical cell. Figure 2a–e specifically display the connection interface between PBSCF_x_ and GDC. Figure 2f shows the cross-section of NiO + GDC|GDC|PBSCF_0.05_ of a single cell. The cathode material of PBSCF_x_ demonstrated good compatibility with the electrolyte and anode of GDC, as well as good contact without delamination. The good compactness of the GDC electrolyte was observed in the electrolyte region. Figure 2g shows the EDS element distribution diagram of the PBSCF_0.05_ cathode material. All elements (Pr, Ba, Sr, Co, O, and F) in PBSCF_0.05_ were uniformly distributed without obvious elemental segregation, and the results were consistent with the chemical formula shown in Table 2. F element was successfully doped into the lattice of PBSC perovskite oxide, which was consistent with the conclusion obtained by XRD.

**Figure 3:**
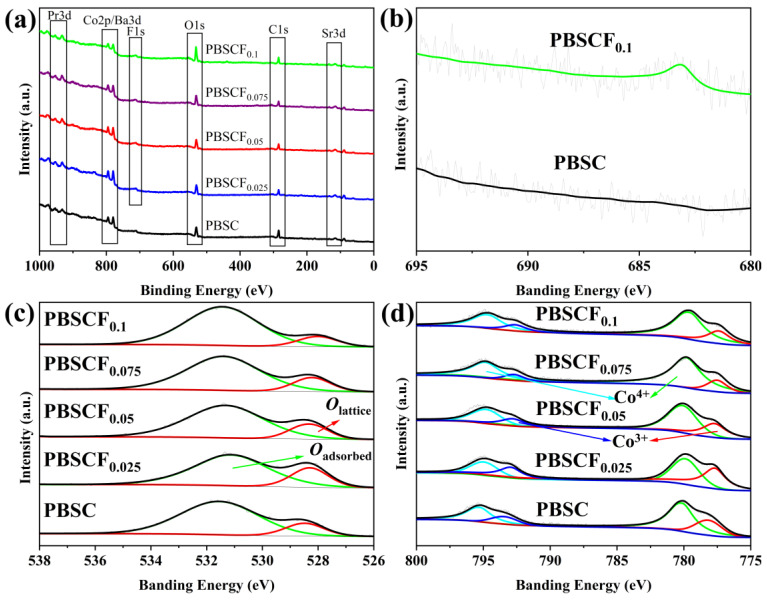
(**a**) XPS full spectrum of PBSCF_x_ series cathode materials; (**b**) F 1s; (**c**) O 1s; (**d**) Co 2p.

To explore whether F was successfully doped into the lattice of PBSCF_x_ cathode materials, we performed XPS analysis on all samples. Figure 3a shows the XPS full-spectrum analysis of the PBSCF_x_ series cathode materials. We observed binding energy peaks of corresponding orbitals from elements Pr, Ba, Sr, Co, F, and O. The position and proportion of binding energy of each element are shown in Table 3. The XPS peaking diagram of F1s in Figure 3b reveals a peak of the cathode material PBSCF_x_ at a binding energy of 684 eV when the doping amounts were *x* = 0 and 0.1, showing that the valence state of F was −1 [21]. This peak was not detected in the PBSC sample. Analysis of the data in Table 3 revealed a minor discrepancy between the atomic content ratio of O and F compared with the theoretical value, suggesting that F could be successfully introduced into the cathode material via the sol–gel method. Furthermore, as the F content increased, the proportion of O gradually decreased, indicating that F successfully substituted a portion of oxygen. As shown in Figure 3c, peak division operation was carried out for O1s of PBSCF_x_ material, and the results showed that O1s revealed two peaks: one at a low binding energy corresponding to *O_lattice_* and another at a high binding energy corresponding to *O_adsorbed_* [22]. The increase in oxygen binding energy indicated a reduction in the Coulombic interaction between the B-site metal ion and O^2−^ ion, thereby improving the reactivity of lattice oxygen and reducing the activation energy required for the migration of the O^2−^ ion [20]. Table 4 shows that an appropriate amount of F doped into the O site could enhance the adsorption of oxygen. Figure 3d shows the XPS data of Co2p for PBSCF_x_ and the sub-peak fitting results. The XPS signal of Co2p showed two peaks of high binding energy and low binding energy, corresponding to Co2p_1/2_ and Co2p_3/2_, respectively. The Co2p_1/2_ peaks were classified as Co^3+^ and Co^4+^ according to the binding energies of 792.9 ± 0.5 and 794.8 ± 0.5 eV, respectively. The Co2p_3/2_ peaks were classified as Co^3+^ and Co^4+^ according to the binding energies of 777.5 ± 0.5 and 779.6 ± 0.5 eV, respectively [23]. The specific location of binding energy and content are listed in Table 5. An appropriate increase in the F content results in an increase in Co^3+^ levels and a decrease in the Co^4+^ content. The increase in Co^3+^ can improve the electrochemical properties of materials.

**Figure 4:**
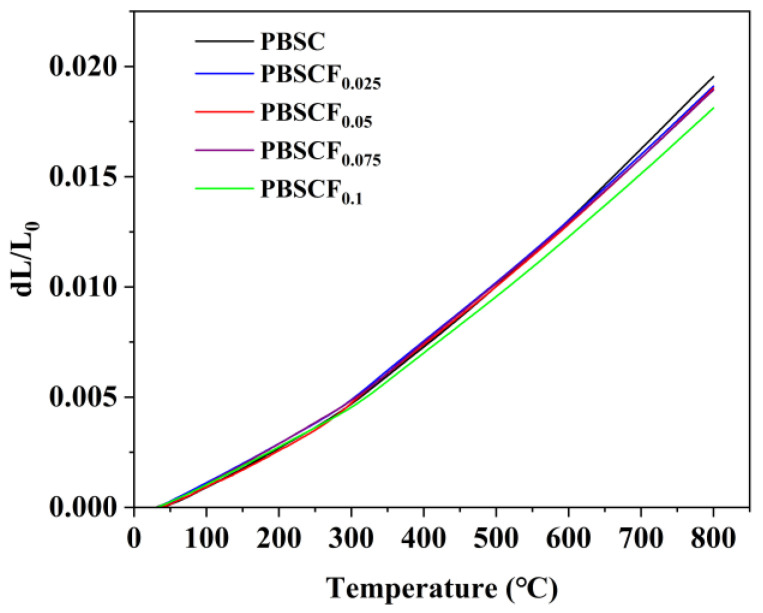
Thermal expansion curve of PBSCF_x_ at air atmosphere.

Figure 4 shows the thermal expansion curves of the PBSCF_x_ series cathode materials measured in argon atmosphere at 30–800 °C. These curves gradually deviated with the increase in temperature, which may be caused by the accelerated release of lattice oxygen [24]. The average TEC of the PBSCF_x_ series cathode materials was calculated according to the thermal expansion curve, and the data are listed in Table 6. As shown in Table 6, with the increase in F doping, the average TEC gradually decreased, and the doping of F reduced TEC from 25.3699 × 10^−6^ K^−1^ of PBSC to 23.5295 × 10^−6^ K^−1^ of PBSCF_0.1_, which was 7.25% lower than the TEC of the sample without F doping. For Co-based perovskite oxides, their chemical expansion is mainly due to the predominance of Co in a low spin state at low temperatures, and Co^n+^ reduction results from the transition of Co to a high spin state or the release of lattice oxygen as temperature rises [25,26]. Nonetheless, appropriate F doping can effectively inhibit the reduction of Co^n+^, reducing the formation of oxygen vacancies and effectively decreasing the TEC of PBSCF_x._ Thus, the thermal stability of the material improved.

**Figure 5:**
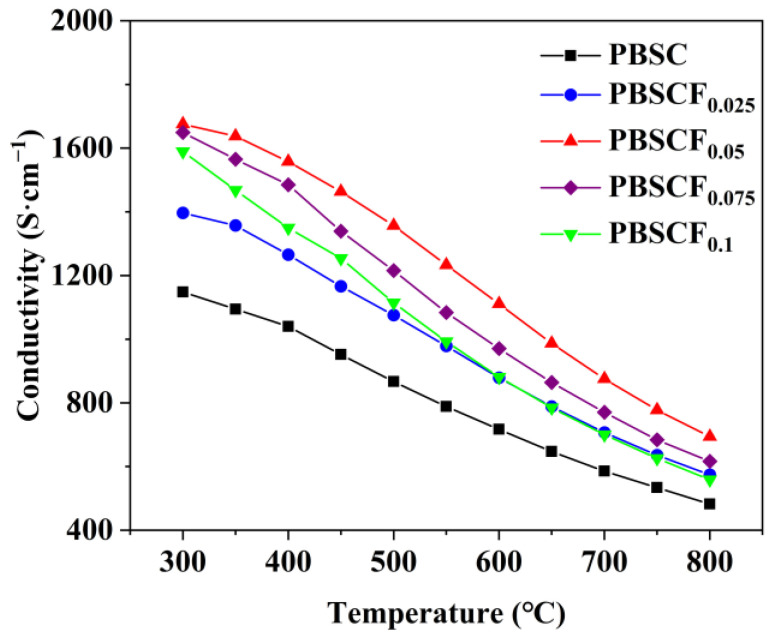
Conductivity of PBSCF_x_ at air atmosphere.

Figure 5 shows how the conductivity of the PBSCF_x_ samples changed with temperature. The conductivity of all PBSCF_x_ series cathode materials decreased linearly with the increase in temperature, showing metal-like conductive behavior. The electrons were mainly transferred along the Co-O-Co layer. At high temperature, the thermal reduction reaction of Co^4+^ led to additional oxygen vacancies in the crystal, and excess oxygen vacancies hindered electron transportation, so conductivity was reduced [27,28]. The defect equation is shown in Equation (1), where $Co_{Co}^{•}$, $Co_{Co}^{\times }$, $O_{O}^{\times }$, and $V_{O}^{••}$ represent Co^4+^, Co^3+^, lattice oxygen, and oxygen vacancy, respectively.

(1) $$2Co_{Co}^{•}+O_{O}^{\times }\Leftrightarrow 2Co_{Co}^{\times }+V_{O}^{••}+\frac{1}{2}O_{2}$$

When the doping amount of F in PBSCF_x_ series samples was 0.05, the conductivity was the highest, indicating that the appropriate amount of doping increased the electron hole concentration. By contrast, when the doping amount was greater than 0.05, the conductivity decreased probably because part of oxygen was released from the lattice after F replaced O; this phenomenon produced a large number of oxygen vacancies, inhibiting electron transfer and increasing conductivity.

**Figure 6:**
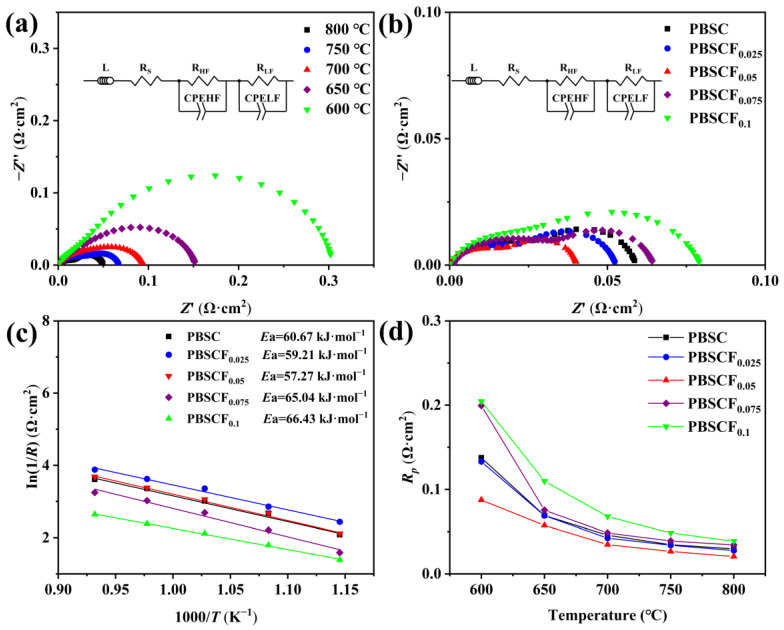
(**a**) Nyquist curve of PBSCF_0.05_|GDC|PBSCF_0.05_ in the range of 600–800 °C; (**b**) Nyquist curve of PBSCF_x_|CGO| PBSCF_x_ at 800 °C; (**c**) *R*_p_ Arrhenius diagram of PBSCF_x_ in the range of 600–800 °C; (**d**) the ASR curve of PBSCF_x_ series cathode materials as a function of temperature.

Electrochemical impedance spectroscopy (EIS) was performed to test the electrochemical impedance of the PBSCF_x_ series cathode materials under air atmosphere, and equivalent circuits *R_s_* (*R*_1_//CPE1, *R*_2_//CPE2) were used to analyze the EIS data, where *R_s_*, *R*_1_, and *R*_2_ represent ohm, high-frequency resistance, and low-frequency resistance, respectively. *R*_1_ is the resistance of the electrochemical reaction at the electrode–electrolyte interface, and *R*_2_ is related to oxygen adsorption/desorption [29]. The polarization resistance is *R_p_* = *R*_1_ + *R*_2_. Figure 6a shows the electrochemical impedance of the symmetrical cell PBSCF_x_|GDC|PBSCF_x_ at 600–800 °C. The impedance of the PBSCF_x_ cathode materials gradually decreased with the increase in temperature. The extra oxygen vacancy generated with the increase in temperature reduced the resistance of the cathode–electrolyte interface and oxygen adsorption/dissociation. Thus, the polarization resistance of the cathode material decreased [30]. Figure 6b,c illustrate that the impedance of the PBSCF_x_ series samples was the smallest when the F content was 0.05, which may be due to the change in oxygen defect concentration caused by the doping of an appropriate amount of F, increasing the oxygen vacancy concentration of the electrode material. The results showed that the activation energy for oxygen adsorption was diminished, and the diffusion ability of oxygen ions was enhanced after F substituted O, which is beneficial for the ORR and consistent with the results of XPS analysis. Figure 6d shows that the area-specific resistance (ASR) values of PBSCF_0.025_ and PBSCF_0.05_ obtained by normalized calculation were 0.0277 and 0.0207 Ω·cm^2^, respectively. Compared with the ASR of PBSC at 800 °C (0.0298 Ω·cm^2^), PBSCF_0.025_ and PBSCF_0.05_ exhibited reductions of approximately 7.05% and 30.54%, respectively, and they were lower than those of other Co-based materials reported in the literature (Table 7). Therefore, the optimal level of F doping facilitated a reduction in the impedance of cathode materials and the activation energy of the ORR, promoting oxygen adsorption/desorption and oxygen ion diffusion and improving the electrochemical performance of cathode materials.

**Figure 7:**
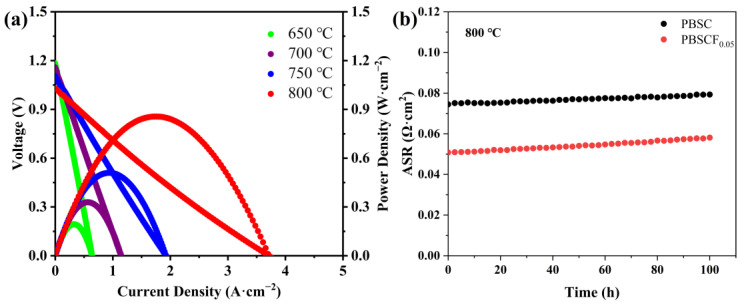
(**a**) I–V–P curve of the single-cell PBSCF_0.05_|GDC|NiO + GDC; (**b**) long-time ASR stability of the symmetrical cells PBSC|LSGM|PBSC and PBSCF_0.05_|LSGM|PBSCF_0.05_ at 800 °C.

The effect of F doping on the electrochemical performance of PBSC cathode materials was characterized by a single cell. A single-cell PBSCF_0.05_|GDC|NiO + GDC was prepared by screen printing. NiO + GDC was used as the fuel electrode, and PBSCF_0.05_ was used as the air electrode. The ambient air was an oxidizer. Figure 7a shows the change in current with voltage and the corresponding power density curve in the temperature range of 800–650 °C with PBSCF_0.05_ as the cathode. The open-circuit voltages at 800 °C, 750 °C, 700 °C, and 650 °C were 1.03, 1.09, 1.15, and 1.18 V, respectively, showing a decrease with the increase in temperature. The peak output power of PBSCF_0.05_ at 800 °C was 856.08 mW·cm^−2^. Figure 7b shows the long-term stability of symmetrical cell PBSC|LSGM|PBSC and PBSCF_0.05_|LSGM|PBSCF_0.05_ at 800 °C for 100 h. ASR of PBSC and PBSCF_0.05_ increased slightly. The doped cathode material had a small ASR, which indicated that the performance of the cathode material was slightly decayed after long-time operation. Therefore, on the basis of the electrochemical performance test results of symmetric cells and single cells, F doping could effectively improve the oxygen reduction activity of PBSC. The reason for the improvement was related to the enhancement of electrical conductivity and thermal matching.

## 3. Experimental

### 3.1. Material Preparation

PrBa_0.8_Sr_0.2_Co_2_O_5+δ−x_F_x_ (*x* = 0, 0.025, 0.05, 0.075, 0.1) series cathode powders were prepared by the sol–gel method. Pr(NO_3_)_3_·6H_2_O (99.99%), Ba(NO_3_)_2_ (A.R.), Sr(NO_3_)_2_ (A.R.), and Co(NO_3_)_2_·6H_2_O (A.R.) were uniformly stirred and dissolved in deionized water according to the stoichiometric ratio. SrF_2_ was dissolved in an appropriate amount of nitric acid solution and added to the above solution, followed by the mixed solution of ammonia and EDTA. The mixture was stirred evenly and added with citric acid (molar ratio of metal ion, citric acid, and EDTA was 1:1:1.5). After stirring for 1 h, ammonia water was added to adjust the pH of the solution to 7–8. It was then stirred in a water bath at 80 °C to form gel and finally heated until the gel spontaneously combusted to yield a fluffy black precursor. The cathode powder was obtained after the precursor was kept at 1100 °C in a muffle furnace for 5 h. According to different F contents, the cathode powder was named PBSCF_x_ (*x* = 0, 0.025, 0.05, 0.0752, 0.1).

### 3.2. Cell Preparation

The electrolyte GDC (Gd_0.1_Ce_0.9_O_1.95_) was pressed into an electrolyte sheet with a diameter of 12 mm by a circular mold. After holding the electrolyte at 1450 °C for 5 h, the cathode paste was evenly applied to both sides of the electrolyte GDC via screen printing. The symmetric battery was obtained by holding the electrolyte at 1100 °C for 5 h. The long-time stability test was conducted using a symmetrical cell prepared with LSGM(La_0.8_Sr_0.2_Ga_0.8_Mg_0.2_) as the electrolyte, and the preparation method was the same as that of the symmetrical cell with GDC as the electrolyte.

A half-cell supported by the anode NiO was prepared by co-pressure. The anode and electrolyte were evenly spread in a circular mold at a pressure of 200 MPa and then held at 1450 °C for 5 h to obtain a half-cell. The cathode paste was uniformly coated on the electrolyte side of the half-cell by screen printing, and the single cell was finally prepared.

### 3.3. Sample Characterization and Performance Test

The cathode powder was detected and analyzed by X-ray diffraction (XRD, Malvern Panalytical, Empyrean, Worcestershire WR14 1XZ, UK) equipped with Cu Kα radiation (40 kV, 40 mA, λ = 1.5418 Å) in the range of 10–80° at the rate of 5°/min and then refined by Rietveld GSAS/EXPGUI software (PC-GSAS NIST (ftp://ftp.ncnr.nist.gov/pub/cryst/gsas/gsas+expgui.exe)). Field-emission scanning electron microscopy (SEM, TESCAN, GAIA3, Brno, Czechia) was performed to observe the cross-sectional microstructure of symmetric cells and single cells, and elemental distribution was analyzed by transmission electron microscopy (TEM, JEOL, 2100F, Tokyo, Japan). The elemental compositions and valence states of PBSCF_x_ samples were analyzed by X-ray photoelectron spectroscopy (XPS, Thermo Scientific, EscaLab250Xi, Waltham, MA, USA). The thermal expansion coefficient (30–800 °C) of cathode materials was measured by a thermal dilatometer (NETZSCH, DIL402C) in argon atmosphere. An electrochemical workstation (Autolab, PGSTAT302N, Chișinău, Moldova) was used to test the conductivity of the material at 300–800 °C in an air atmosphere via the DC four-probe method and assess the electrochemical impedance in an air atmosphere in the range of 0.1 Hz–100 kHz (600–800 °C). The power output of a single cell was tested at 50 °C intervals within the range of 650–800 °C. The long-time stability of the symmetrical cells PBSC and PBSCF_0.05_ at 800 °C for 100 h was tested at intervals of 2.5 h.

## 4. Conclusions

In summary, this study successfully prepared pure tetragonal PBSCF_x_ (*x* = 0, 0.025, 0.05, 0.075, 0.1) series cathode materials by the sol–gel method, and the lattice gradually shrank with the increase in F^−^ doping amount. The conductivity of PBSC and PBSCF_0.05_ at 300 °C was 1148.29 and 1675.61 S·cm^−1^, respectively, which was 45.92% higher than that of undoped samples. An appropriate amount of F^−^ doping could limit Co^n+^ to the intermediate spin state, thereby reducing the TEC of the cathode material. The 25.3699 × 10^−6^ K^−1^ of PBSC was reduced to 23.5295 × 10^−6^ K^−1^ of PBSCF_0.1_, indicating that an appropriate amount of F^−^ doping could improve thermal matching between the cathode material and the electrolyte. The ASR values of the symmetric cells prepared with PBSC and PBSCF_0.05_ as cathode at 800 °C were 0.0298 and 0.0207 Ω·cm^2^, respectively, and the ASR values of the optimized samples were reduced by 30.54%. The results showed that F doping could reduce the activation energy and accelerate the transfer of oxygen ions. The anodized single-cell PBSCF_0.05_|GDC|NiO + GDC had a power density of 856.08 mW·cm^−2^ at 800 °C. The symmetrical cells PBSC|LSGM|PBSC and PBSCF_0.05_|LSGM|PBSCF_0.05_ had slight decay at 800 °C. By weakening the chemical bond between metal cation and O^2−^, F-doping promoted the diffusion of oxygen in the cathode and the surface adsorption process, thereby improving the reaction kinetics of the rate-limiting step and enhancing the electrochemical performance of the material. Thus, F-doped PrBa_0.8_Sr_0.2_Co_2_O_5+δ_ cathode materials showed good electrochemical properties at medium temperature, and can be a candidate material for the development of high-performance and high-activity perovskite cathode.

## Figures and Tables

**Table 1:** Cell parameters of the cathode material PBSCF_x_.

PBSCF*_x_*	Space Group	a (Å)	c (Å)	V (Å)	χ^2^	R_wp_ (%)
*x* = 0	P4/mmm	3.91980	7.73349	118.824	1.24	7.532
*x* = 0.025	P4/mmm	3.91566	7.72752	118.481	1.36	7.596
*x* = 0.05	P4/mmm	3.91282	7.71982	118.192	1.26	7.389
*x* = 0.075	P4/mmm	3.91050	7.70556	117.834	1.25	7.973
*x* = 0.1	P4/mmm	3.90391	7.69884	117.334	1.32	8.117

**Table 2:** TEM-EDS testing of PBSCF_0.05_ cathode material to determine the proportion of each element.

Atom	Atomic Percentage (%)
O	55.07
Co	23.27
Pr	10.52
Ba	8.29
Sr	2.23
F	0.62

**Table 3:** Full-spectrum XPS results of PBSCF_x_ series cathode materials (unit: eV (%)).

Sample	O1s (eV (%))	Ba3d (eV (%))	Co2p_1/2_ (eV (%))	Co2p_3/2_ (eV (%))	Sr3d (eV (%))	F1s (eV (%))	Pr3d (eV (%))
PBSC	531.11	779.33	794.65	779.28	133.15	0(0)	932
	(33.96)	(3.56)	(7.97)	(6.49)	(1.83)		(7.23)
PBSCF_0.025_	530.59	779.34	794.69	779.30	132.83	683	931.62
	(33.79)	(4.02)	(9.86)	(7.63)	(2.53)	(0.17)	(6.45)
PBSCF_0.05_	530.95	779.54	794.79	779.50	133.11	684.56	932
	(33.42)	(3.48)	(8.53)	(7.80)	(3.66)	(0.34)	(6.52)
PBSCF_0.075_	531.19	779.39	794.78	779.35	133	684.66	931.28
	(32.89)	(3.29)	(9.67)	(8.30)	(3)	(0.5)	(6.35)
PBSCF_0.1_	531.81	779.32	794.78	779.26	133.59	684.74	929.09
	(32.49)	(3.20)	(8.35)	(6.43)	(3.45)	(0.66)	(6.65)

**Table 4:** O1s peak fitting results of XPS spectra of PBSCF_x_ series cathode materials (unit: eV (%)).

Sample	Lattice Oxygen (eV (%))	Adsorbed Oxygen (eV (%))
PBSC	528.48 (5.90)	531.54 (23.50)
PBSCF_0.025_	528.30 (7.90)	531.12 (23.04)
PBSCF_0.05_	528.32 (5.46)	531.32 (25.07)
PBSCF_0.075_	528.22 (5.93)	531.40 (27.75)
PBSCF_0.1_	528.01 (5.64)	531.44 (25.74)

**Table 5:** Co2p peak fitting results of XPS spectra of PBSCF_x_ series cathode materials (unit: eV (%)).

Sample	${\mathrm{Co}}_{2p1/2}^{4+}$	${\mathrm{Co}}_{2p1/2}^{3+}$	${\mathrm{Co}}_{2p3/2}^{4+}$	${\mathrm{Co}}_{2p3/2}^{3+}$
PBSC	795.27 (9.31)	793.44 (1.69)	780.14 (9.78)	778.18 (1.66)
PBSCF_0.025_	794.95 (8.65)	792.96 (2.22)	779.90 (8.71)	777.71 (2.22)
PBSCF_0.05_	794.78 (6.58)	792.81 (3.28)	780.09 (6.11)	777.52 (3.04)
PBSCF_0.075_	794.86 (7.63)	792.70 (2.54)	779.84 (8.11)	777.52 (1.85)
PBSCF_0.1_	794.64 (7.86)	792.62 (2.43)	779.59 (8.32)	777.40 (1.19)

**Table 6:** Average TEC of PBSCF_x_ series cathode materials at 30–800 °C.

Sample	TEC (×10^−6^ K^−1^)
PBSC	25.3699
PBSCF_0.025_	24.8132
PBSCF_0.05_	24.6866
PBSCF_0.075_	24.5928
PBSCF_0.1_	23.5295

**Table 7:** ASR values of Co-based materials reported in other literature.

Sample	Electrode	Temperature (°C)	ASR (Ω·cm^2^)	Reference
Sm_0.7_Ca_0.3_BaCo_2_O_5+*δ*_	SDC	700	0.075	[31]
LaBa_0.5_Sr_0.25_Ca_0.25_Co_2_O_5+δ_	SDC	800	0.075	[32]
PrBa_0.94_Co_2_O_5+*δ*_	GDC	700	0.025	[33]
Gd_1.05_Ba_1.05_Co_2_O_5+*δ*_	GDC	800	0.146	[34]
Pr_0.94_Ba_0.7_Sr_0.3_Co_2_O_5+_*_δ_*	GDC	700	0.031	[15]
PrBa_0.8_Sr_0.2_Co_2_O_4.95+*δ*_F_0.05_	GDC	800	0.0207	this work

## Data Availability

The data presented in this study are available on request from the corresponding author.
